# Transcriptional analysis of oligosaccharide utilization by *Bifidobacterium lactis* Bl-04

**DOI:** 10.1186/1471-2164-14-312

**Published:** 2013-05-10

**Authors:** Joakim M Andersen, Rodolphe Barrangou, Maher Abou Hachem, Sampo J Lahtinen, Yong Jun Goh, Birte Svensson, Todd R Klaenhammer

**Affiliations:** 1 Enzyme and Protein Chemistry, Department of Systems Biology, Technical University of Denmark, Søltofts Plads Building 224, Kgs. Lyngby DK-2800, Denmark; 2 Department of Food, Bioprocessing and Nutrition Sciences, North Carolina State University, Raleigh NC 27695, USA; 3 DuPont Nutrition and Health, 3329 Agriculture Drive, Madison WI 53716, USA; 4 DuPont Nutrition and Health, Sokeritehtaantie 20, Kantvik FI-02460, Finland

**Keywords:** *Bifidobacterium lactis*, Transcriptomics, ABC transporter, GPH transporter, Prebiotics, Glycoside hydrolase

## Abstract

**Background:**

Probiotic bifidobacteria in combination with prebiotic carbohydrates have documented positive effects on human health regarding gastrointestinal disorders and improved immunity, however the selective routes of uptake remain unknown for most candidate prebiotics. The differential transcriptomes of *Bifidobacterium animalis* subsp. *lactis* Bl-04, induced by 11 potential prebiotic oligosaccharides were analyzed to identify the genetic loci involved in the uptake and catabolism of α- and β-linked hexoses, and β-xylosides.

**Results:**

The overall transcriptome was modulated dependent on the type of glycoside (galactosides, glucosides or xylosides) utilized. Carbohydrate transporters of the major facilitator superfamily (induced by gentiobiose and β-galacto-oligosaccharides (GOS)) and ATP-binding cassette (ABC) transporters (upregulated by cellobiose, GOS, isomaltose, maltotriose, melibiose, panose, raffinose, stachyose, xylobiose and β-xylo-oligosaccharides) were differentially upregulated, together with glycoside hydrolases from families 1, 2, 13, 36, 42, 43 and 77. Sequence analysis of the identified solute-binding proteins that determine the specificity of ABC transporters revealed similarities in the breadth and selectivity of prebiotic utilization by bifidobacteria.

**Conclusion:**

This study identified the differential gene expression for utilization of potential prebiotics highlighting the extensive capabilities of *Bifidobacterium lactis* Bl-04 to utilize oligosaccharides. Results provide insights into the ability of this probiotic microbe to utilize indigestible carbohydrates in the human gastrointestinal tract.

## Background

Health-promoting microbes, defined as probiotics [1], have gained increased interest for use in food and dietary supplement applications to improve health and well being. Clinical research has shown that bifidobacteria are an important genus for probiotic interventions through clinical studies [2,3]. Benefits reported using bifidobacteria include improvement of bowel functions [4], prevention of necrotizing enterocolitis in infants [5], treatment of Crohn’s disease [6] and modulation of immune functions in the elderly [7]. Understanding the mechanisms of action underlying the probiotic attribute of bifidobacteria on the molecular level has been restricted to functional extrapolation from genome sequencing [8]. Interestingly, from the 53 *Bifidobacterium* genomes that have been deposited publicly to date, comparative analysis has revealed the genetic diversity of bifidobacteria [9], leading to identification of genetic loci for colon adaptation by host mucin degradation in *B. bifidum*[10], foraging of dietary carbohydrates in e.g. *B. longum*[11] or the important feature of human milk utilization [12] enabling colonization of the infant GIT [11,13,14] .

Enhancement of beneficial microbes within the gastrointestinal tract (GIT) can be achieved by providing selectively utilizable carbohydrates [15], defined as prebiotics [16]. Prebiotics are dietary carbohydrates, resistant to the host digestive system and main commensal microbiota residing in the colon. To date, only a few carbohydrates have been documented as prebiotics, namely β-galactooligosaccharides (GOS), lactulose, fructo-oligosaccharides and inulin [17]. Several candidate prebiotics have been proposed, but there is a need for additional studies that document their selective utilization by beneficial microbes within the human GIT [18,19]. The diversity of prebiotics with respect to size, composition and glycosidic linkages require a multitude of transporters and hydrolytic enzymes, some of which are predicted to occur widely within bifidobacteria, based mostly on *in silico* analysis [20,21].

*Bifidobacterium animalis* subsp. *lactis* has been reported to exert positive effects as a probiotic microbe in clinical studies [22,23], or when supplemented as a synbiotic in combination with prebiotics [24]. The annotated genome sequence of *B. lactis* Bl-04 revealed putative prebiotic transport and catabolic pathways, suggesting the bacterium to be highly adapted to the GIT and capable of utilization of dietary-derived complex oligosaccharides [25]. In the present study, we used differential transcriptomics to identify genetic loci encoding uptake and hydrolytic pathways of potential prebiotics manifested by 11 structurally diverse galactosides, glucosides and xylosides within *B. lactis* Bl-04. This work validated and expanded the tentative *in silico* predictions of oligosaccharide transporters and specificities of glycoside hydrolases.

Furthermore the perspective of the study enables the combination of transcriptomics and genome mining to serve as a platform for future functional work within prebiotic utilization by bifidobacteria.

## Results

### Oligosaccharide induced global transcriptome profile of *B. lactis* BL-04

**Figure 1:**
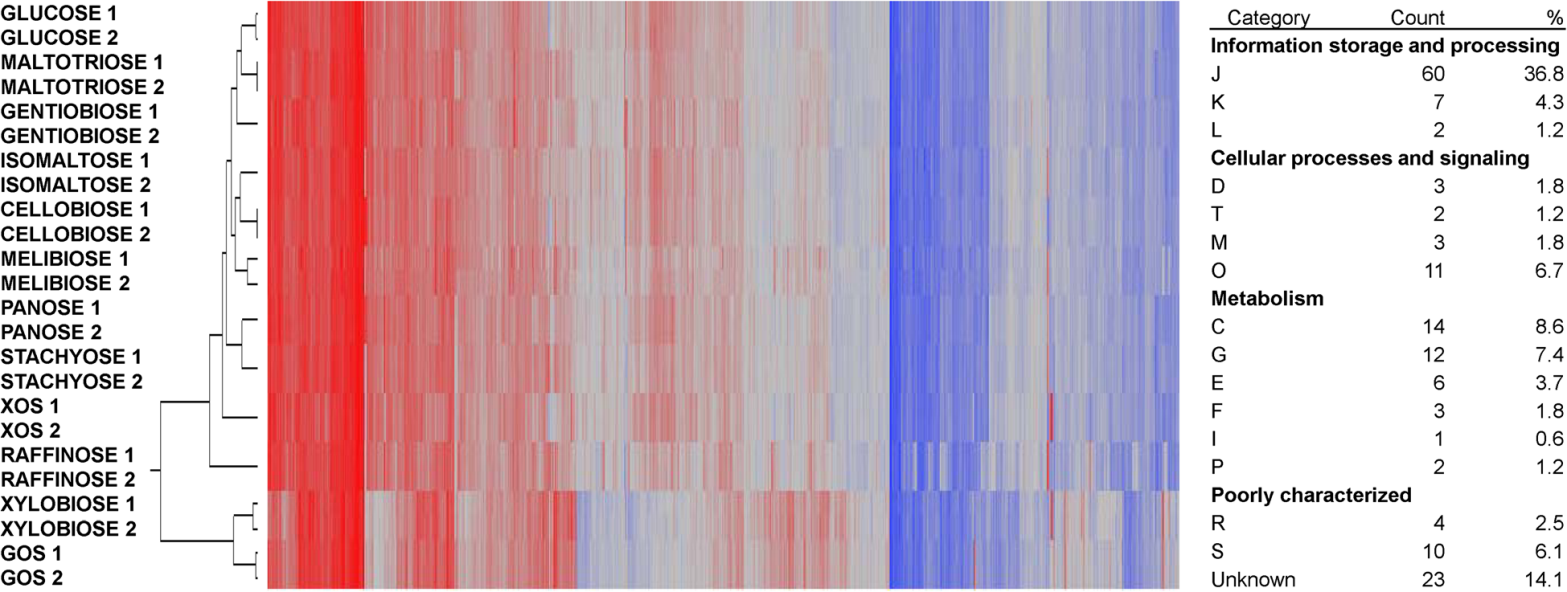
**Two-way clustering of the global gene expression profiles and COG distribution of constitutively expressed genes.** Gene expression intensities are represented by red coloring: up-regulation, blue coloring: down-regulation. Technical replicates for each carbohydrate are numbered and showed overall high reproducibility. The highest expressed decile (163 genes) of the global transcriptome across all carbohydrates conditions was assigned COG categories (assigned both as numbers and percentages), highlighting essential metabolic pathways of *B. lactis* Bl-04.

Global gene expression profiles were obtained for *B. lactis* Bl-04, exponentially growing on 11 potential prebiotic oligosaccharides and glucose (Table 1), representing α-galactosides (melibiose, raffinose and stachyose), β-galactosides (GOS), α-glucosides (isomaltose, maltotriose and panose), β-glucosides (cellobiose and gentiobiose) and β-xylosides (xylobiose and xylo-oligosaccharides (XOS)). Growth of *B. lactis* Bl-04 on various mono, di and oligosaccharides were previously published [26]. The gene expression levels were quantified by whole genome DNA microarrays showing an overall comparable gene expression profiles across all 12 carbohydrate treatments and with high technical reproducibility (Figure 1). Only a subset of genes were upregulated differentially in response to each oligosaccharide, although a slight deviation of the GOS and xylobiose samples was observed. The 10 % of the highest constitutively expressed genes for all carbohydrate treatments (163 genes) were assigned Clusters of Orthologous Groups (COG) categories [27] and emphasized main cellular functions of growth and energy turn-over (Figure 1). Notably, of the highly expressed single genes (listed by *B. lactis* Bl-04 locus tag numbers), several highlight molecular functions related to probiotic mechanisms in *B. lactis* putatively involved in fibronectin adhesion (Balac_1484–1485), host plasminogen interactions (Balac_1017 and Balac_1557), phage immunity [28] (Balac_1305), bile salt hydrolysis (Balac_0863) and peroxide reduction (Balac_0865). In addition, part of an oligosaccharide ATP-binding cassette (ABC) transporter encoding a solute-binding protein (SBP) (Balac_1565), and an ATP-binding protein associated with oligosaccharide uptake by ABC transporters (Balac_1610) were both found, linking catabolic adaptation to the primary physiological functions of *B. lactis* Bl-04. These findings correlate with previously proposed molecular functions related to probiotic mechanisms in *B. lactis*[29].

**Table 1:** Carbohydrates, used for DNA microarray studies, listed with glycoside structure and type, supplier and purity

**Carbohydrate**	**Structure**^**1**^	**Glycoside type**	**DP**^**2**^	**Manufacturer or supplier**	**Purity (as given by Manufacturer or supplier)**
Glucose	Glc*p*	glucoside	1	Sigma	> 99%
GOS	[β-d-Gal*p*-[1]–[4]]_n_-d-Glc*p*	galactoside	2–6	Dupont	> 94% DP ≥ 2
Melibiose	α-d-Gal*p*-[1]–[6]-d-Glc*p*	galactoside	2	Sigma	> 98%
Raffinose	α-d-Gal*p*-[1]–[6]-d-Glc*p*-(α1,β2)-D-Fru*f*	galactoside	3	Sigma	> 99%
Stachyose	[α-d-Gal*p*-[1]–[6]]_2_-d-Glc*p*-(α1,β2)-d-Fru*f*	galactoside	4	Sigma	> 98%
Isomaltose	α-d-Glc*p*-[1]–[6]-d-Glc*p*	glucoside	2	Sigma	> 98%
Panose	α-d-Glc*p*[1]–[6]-α-d-Glc*p-*[1]–[4]-d-Glc*p*	glucoside	3	Sigma	> 98%
Maltotriose	α-d-Glc*p-*[1]–[4]-α-d-Glc*p-*[1]–[4]-d-Glc*p*	glucoside	3	Dupont	> 95%
Cellobiose	β-d-Glc*p*-[1]–[4]-d-Glc*p*	glucoside	2	Fluka AG	> 99%
Gentiobiose	β-d-Glc*p*-[1]–[6]-d-Glc*p*	glucoside	2	Sigma	> 98%
Xylobiose	β-d-xyl*p*-[1]–[4]-d-xyl*p*	xyloside	2	Dupont	> 95%
XOS	[β-d-xyl*p*-[1]–[4]]_m_-d-xyl*p*	xyloside	2–7	Shandong Longlive Bio-technology Co., Ltd, (China)	> 90%^3^

^1^n = 1–5 as previously described [70], m =1–6 as stated by manufacturer.

^2^Degree of polymerization.

^3^The XOS composition and purity was previously determined [60].

**Figure 2:**
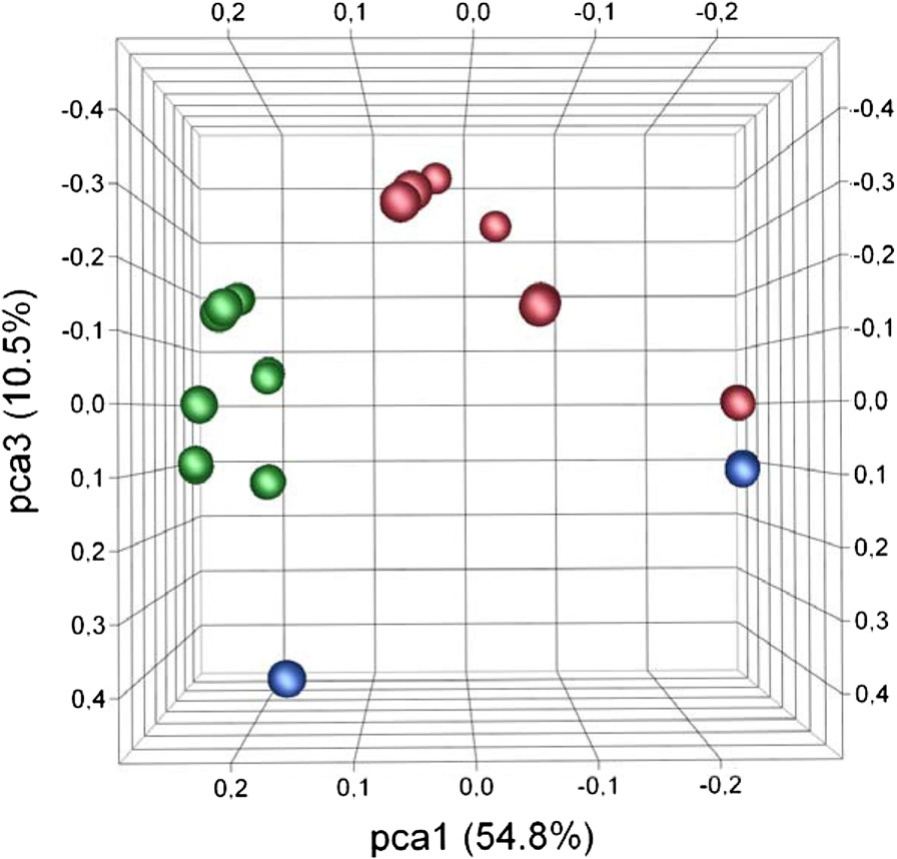
**Principal component analysis of the global transcriptome for *B. lactis *Bl-04 cultivated with prebiotics showing differentiation of the global gene expression profiles depending on the glycoside type of carbohydrates utilized (pca2 = 22.2%).** Galactosides in red (GOS, melibiose, raffinose, stachyose), glucosides in green (cellobiose, gentiobiose, glucose, maltotriose, isomaltose, panose) and xylosides in blue (XOS, xylobiose).

Functional grouping of global gene expression was observed based on the type of glycoside utilized (galactosides, glucosides or xylosides) from principal component analysis (Figure 2). A clear differentiation of the expressed global transcriptome was observed based on the type of glycoside utilized, indicating that prebiotics can affect the global transcriptome, and therefore physiological functions in *B. lactis* Bl-04. Furthermore, specific genetic loci were significantly differentially regulated by specific carbohydrates, which indicates their potential involvement in the uptake and catabolism of the respective glycosides (Figure 1).

### Differentially upregulated genes conferring potential prebiotic utilization

**Figure 3:**
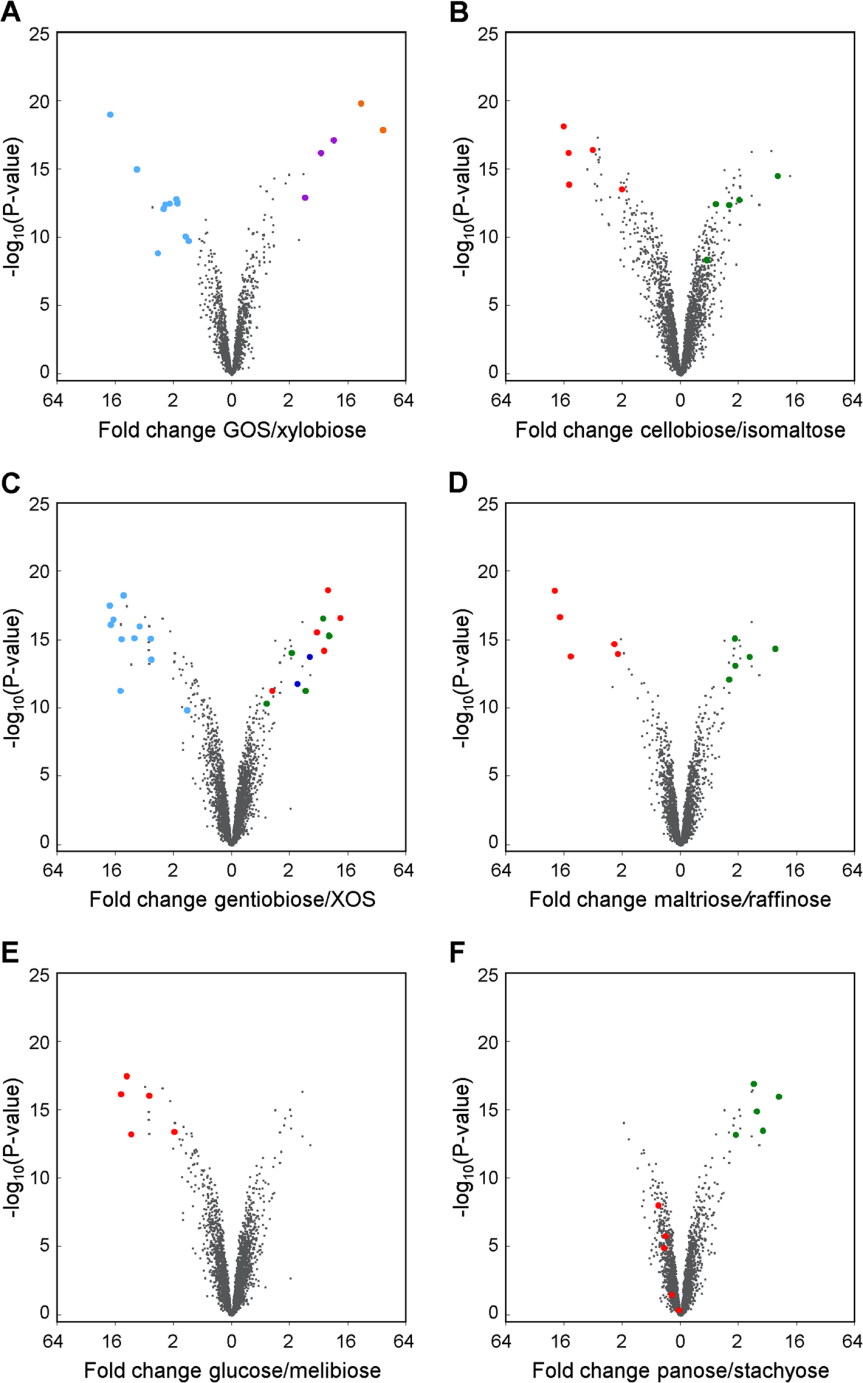
**Representative volcano plots of pairwise comparisons of the oligosaccharide-induced differential global transcriptome in *B. lactis *Bl-04.** All genes are shown by solid grey circles, and putative carbohydrate-active protein encoding genes that were significantly up-regulated are highlighted with solid circles and color-coded by gene cluster and as listed in Table 2: cluster **A** (blue), cluster **B** (orange), cluster **C** (purple), cluster **D** (light blue), cluster **E** (green) and cluster **F** (red).

**Figure 4:**
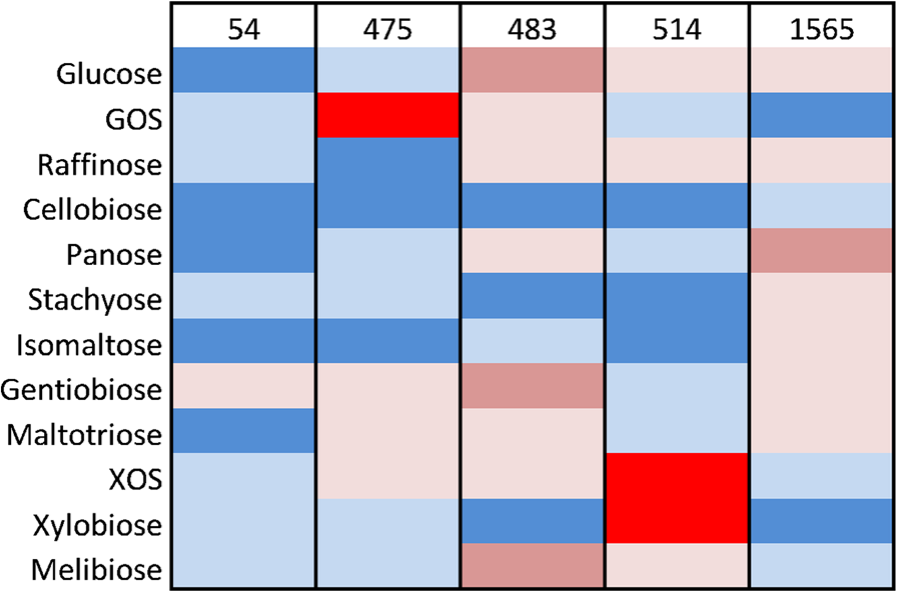
**Heat map representation of RT-qPCR validation of microarray gene expression values.** The mRNA transcript level were quantified for each of the five genes in each of the 12 carbohydrate conditions and color-coded as the relative fold upregulation of each gene: Blue [1–2], light blue [5–9], red [9–17] and strong red [>16].

Analysis of the differential upregulation of specific genes mediating potential prebiotic utilization was conducted by one-way analysis of variance (ANOVA) and visualized by volcano plots (Figure 3) to identify statistically significant genes (cut off: p-value < 10^-8.04^) upregulated by each carbohydrate in the whole genome DNA microarray. An average of 56 genes were more than 2-fold differentially upregulated and above the statistical threshold for each pairwise comparison. Analysis revealed how subsets of genes involved with oligosaccharide metabolism were consistently differentially expressed throughout the ANOVA (Table 2, and Figure 4 for real time quantitative-PCR validation of selected genes). This led to the reconstruction of six putative gene clusters based on the differential upregulation of specific genes to specific oligosaccharide treatments. Gene clusters encoding a transporter and glycoside hydrolase(s) were linked to the uptake and degradation of substrates that varied in the degree of polymerization, glycosidic linkage or monosaccharide composition.

**Table 2:** Statistically significant upregulated genes involved in carbohydrate uptake and catabolism

**ORF**	**Gene annotation**	**Inducing CHO type**	**Volcano plot (Figure**3**)**	**Highest inducing CHO**	**Gene cluster (Figure**5**)**	**Fold upregulated**	**-log**_**10**_**(P-value)**
Balac_0053	β-galactosidase, GH42	Glucoside	C	gentiobiose	A	6.5	13.8
Balac_0054	MFS permease	Glucoside	C	gentiobiose	A	4.8	11.8
Balac_0475	MFS permease	Galactoside	A	GOS	B	21.8	19.9
Balac_0476	β-galactosidase, GH2	Galactoside	A	GOS	B	36.9	17.9
Balac_0484	β-galactosidase, GH42	Galactoside	A	GOS	C	11.4	17.2
Balac_0485	ABC transporter, permease component	Galactoside	A	GOS	C	8.4	16.2
Balac_0486	ABC transporter, permease component	Galactoside	A	GOS	C	5.7	12.9
Balac_0511	Xylose isomerase	xyloside	A,C	XOS	D	13.8	15.1
Balac_0512	α-l-arabinofuranosidase, GH43	xyloside	A,C	XOS	D	6.8	13.6
Balac_0513	Transcriptional regulator (*lacI* type)	xyloside	A,C	Xylobiose	D	3.0	10.1
Balac_0514	ABC transporter, oligosaccharide-binding protein	xyloside	A,C	XOS	D	9.0	16.0
Balac_0515	ABC transporter, permease component	xyloside	A,C	XOS	D	16.8	16.5
Balac_0516	ABC transporter, permease component	xyloside	A,C	XOS	D	18.3	17.5
Balac_0517	β-xylosidase, GH43	xyloside	A,C	XOS	D	17.9	16.1
Balac_0518	Putative carbohydrate esterase	xyloside	A,C	XOS	D	14.2	11.3
Balac_0519	Esterase	xyloside	A,C	XOS	D	6.9	15.1
Balac_0520	α-l-arabinofuranosidase, GH43	xyloside	A,C	XOS	D	10.2	15.2
Balac_0521	Xylulose kinase	xyloside	A,C	Xylobiose	D	18.2	19.0
Balac_1567	4-α-glucanotransferase	glucoside	B,D	Maltriose	E	9.7	14.5
Balac_1569	ABC transporter, permease component	glucoside	B,D	Cellobiose	E	5.2	13.7
Balac_1570	ABC transporter, permease component	glucoside	B,D	Cellobiose	E	3.7	15.1
Balac_1571	Transcriptional regulator (*lacI* type)	glucoside	B,D	Cellobiose	E	3.7	13.1
Balac_1572	ABC transporter, oligosaccharide-binding protein	glucoside	B,D	Cellobiose	E	3.2	12.1
Balac_1593	oligo-1,6-α-glucosidase, GH13	Galactoside, Glucoside	B,D,E	Isomaltose	F	4.5	14.0
Balac_1597	ABC transporter, permease component	Galactoside, Glucoside	B,D,E	Raffinose	F	14.1	13.9
Balac_1598	ABC transporter, permease component	Galactoside, Glucoside	B,D,E	Isomaltose	F	20.1	18.6
Balac_1599	ABC transporter, oligosaccharide-binding protein	Galactoside, Glucoside	B,D,E	Isomaltose	F	17.8	16.7
Balac_1601	α-galactosidase, GH36	Galactoside, Glucoside	B,D,E	Raffinose	F	8.1	16.4

The genes are listed by ascending locus tag numbers with the principle type of glycoside. Only the oligosaccharide that elicited the highest significance level (−log_10_(P-value)) is listed for genes that are upregulated by more than one oligosaccharide.

**Figure 5:**
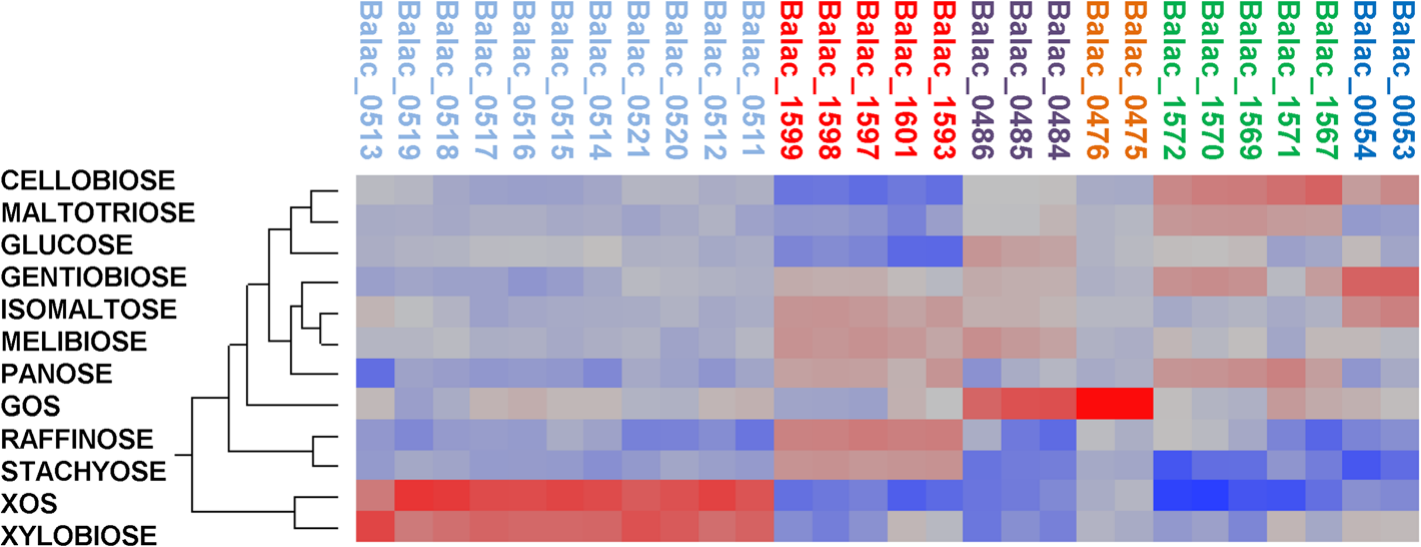
**Two-way clustering of the expression profile for genes identified to be differentially upregulated by ANOVA.** The coloring of each ORF corresponds to the gene cluster of Figure 2 and Table 2.

Moreover, the relative induction of gene clusters involved in carbohydrate uptake and catabolism (Figure 5) strongly supports the identification of the differential specificities of upregulated proteins involved with utilization of the various oligosaccharides.

### Gene cluster analysis and functional assignment

**Figure 6:**
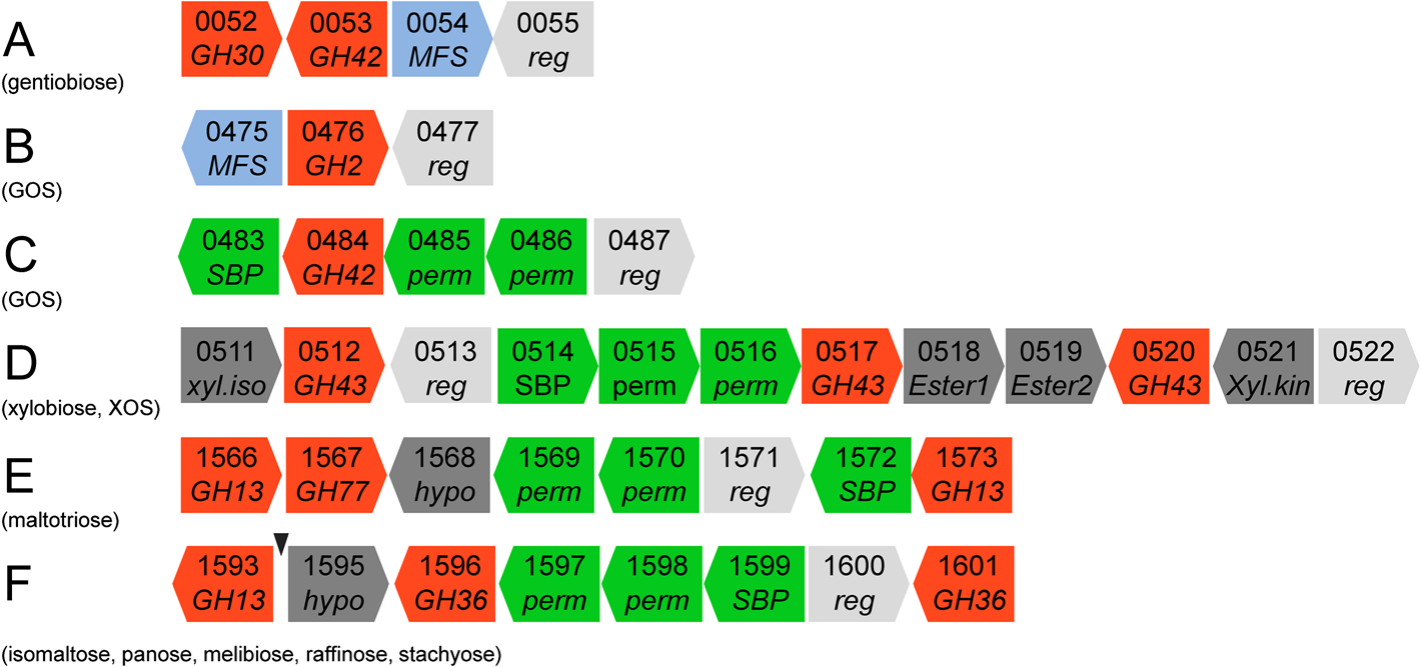
**Organization of differentially expressed gene clusters encoding proteins predicted to be involved with prebiotic utilization.** Genes are listed with locus tag numbers and gene functions are colored as: glycoside hydrolases in red, ABC transporter SBPs- and permeases (perm) in green, MFS transporters in blue, transcriptional regulators (reg) in light grey and hypothetical proteins (hypo), carbohydrate esterases (ester1 and ester2), xylose isomerase (xyl.iso) and xylulose kinase (xyl.kin) all in dark gray. A short putative nonfunctional ORF is highlighted by a black triangle.

Analysis of differentially up-regulated loci enabled the identification of six gene clusters conferring the uptake and hydrolysis of the oligosaccharides used in the study (Figure 6A–F). Common to all the identified loci is that they encoded a transport system, a transcriptional regulator and one or more glycoside hydrolases (GHs) as predicted from the glycoside hydrolase family annotation in the CAZy database [30] (Figure 3). Four ATP-binding cassette (ABC) systems and two major facilitator superfamily (MFS) transporters, including one putative glycoside-pentoside-hexuronide (GPH) system were identified [31], supporting the differential expression of gene clusters being induced by multiple oligosaccharides (Figure 3).

Gene cluster A, differentially up-regulated by gentiobiose, encoded an MFS transporter (Balac_0054), with only 25% amino acid sequence identity to a sucrose permease from *Arabidopsis thaliana* (uniprot: Q9FG00), and an intracellular (as predicted by SignalP 4.0 [32]) putative β-galactosidase of GH42 (Balac_0053), adjacent to a GH30 subfamily 1 (GH30_1) putative β-glucosidase (Balac_0052) [33] not identified from the ANOVA. Interestingly, GH42 enzymes are only reported to be active on β-galactoside linkages [34], whereas GH30_1 enzymes harbor several specificities including endo-β-[1,4]-glucosidases rendering the GH30_1 a likely candidate for gentiobiose hydrolysis. Interestingly, co-occurrence of the GH42 and the GH30_1 genes is also observed in other bifidobacteria e.g. *B. adolescentis* ATCC 15703 and *B. dentium* Bd1. The rationale for the co-occurrence of these two GH genes is currently unclear, but the data identifies a novel route for gentiobiose uptake via the MFS transporter and hydrolysis by the putative GH30_1 family β-[1,4]-glucosidase.

The GOS substrate upregulated the expression of two loci: an MFS transporter (Balac_0475) homologous to the lactose transporter from *B. longum* NCC2705 [35], and a typical GH2 β-galactosidase (Balac_0476, cluster B) and cluster C (Balac_0483–0486) encoding the heterodimeric permease and the solute binding protein of an ABC transport system along with a GH42 putative β-galactosidase. The upregulation of two loci with a similar architecture was also reported in *B. breve*[36].

Xylobiose and XOS induced locus D encoding an ABC transporter, a putative GH43 β-xylosidase (Balac_0517) and two putative GH43 arabinofuranosidases (Balac_0512 and Balac_0520) identified based on homology to characterized bifidobacterial enzymes [37,38]. This suggests that the gene cluster mediates the transport and hydrolysis of both undecorated and arabinosyl-decorated XOS. The gene cluster also encodes a xylose isomerase and a xylulose kinase necessary to convert xylose to 2-xylulose-5 phosphate for entry into the bifid-shunt pathway [39]. The removal of acetyl sidechains that typically occur at the C2 or C3 of mainchain xyloxyl residues and feruloyl esters at the C5 or C2 of arabinosyl decorations in arabinoxylan is a prerequisite for the utilization of decorated arabinoxylo-oligosaccharides [40]. Notably, two putative carbohydrate esterases (Balac_0518 and Balac_0519) were upregulated highlighting the ability of the *B. animlais* subsp. *lactis* to remove acetyl or feruloyl sidechains from imported arabinoxylan fragments.

The organization and type of genes in cluster E showed resemblance to maltose operons from *B. longum* NCC2705 [35]. In the current study, however, the gene cluster was also upregulated by the trisaccharides panose, maltotriose and remarkably the β-linked disaccharide cellobiose. The gene landscape of this maltooligosaccharide gene cluster differed in the types of glycoside hydrolases encoded in the comparison to counterparts reported in other Gram positive bacteria [41]–[43] suggesting divergence in α-glucan metabolism.

An ABC transporter was identified in cluster F and was induced by the raffinose family oligosaccharides (RFO) melibiose, raffinose and stachyose representing α-1,6 linked galactosides, along with the α-1,6 linked glucosides isomaltose and panose. The GH36 subfamily 1 (GH36_1, Balac_1601) α-galactosidase [44] confers the hydrolysis of the α-1,6 linked galactosides, while the GH13 oligo-α-1,6-glucosidase (Balac_1593) catalyzes the hydrolysis of α-1,6 linked glucosides [45]. *B. lactis* Bl-04 encoded a total of three GH36, yet the remaining two (Balac_1537 and Balac_1596) were not differentially expressed. Further analysis of the global transcriptome (Figure 1) showed low basal expression of Balac_1537 in all conditions suggesting that the gene product plays a continuous metabolic role in cell function, while Balac_1596, assigned to GH36_2 (homologous to plant raffinose synthases [44]) was not expressed under these conditions.

**Figure 7:**
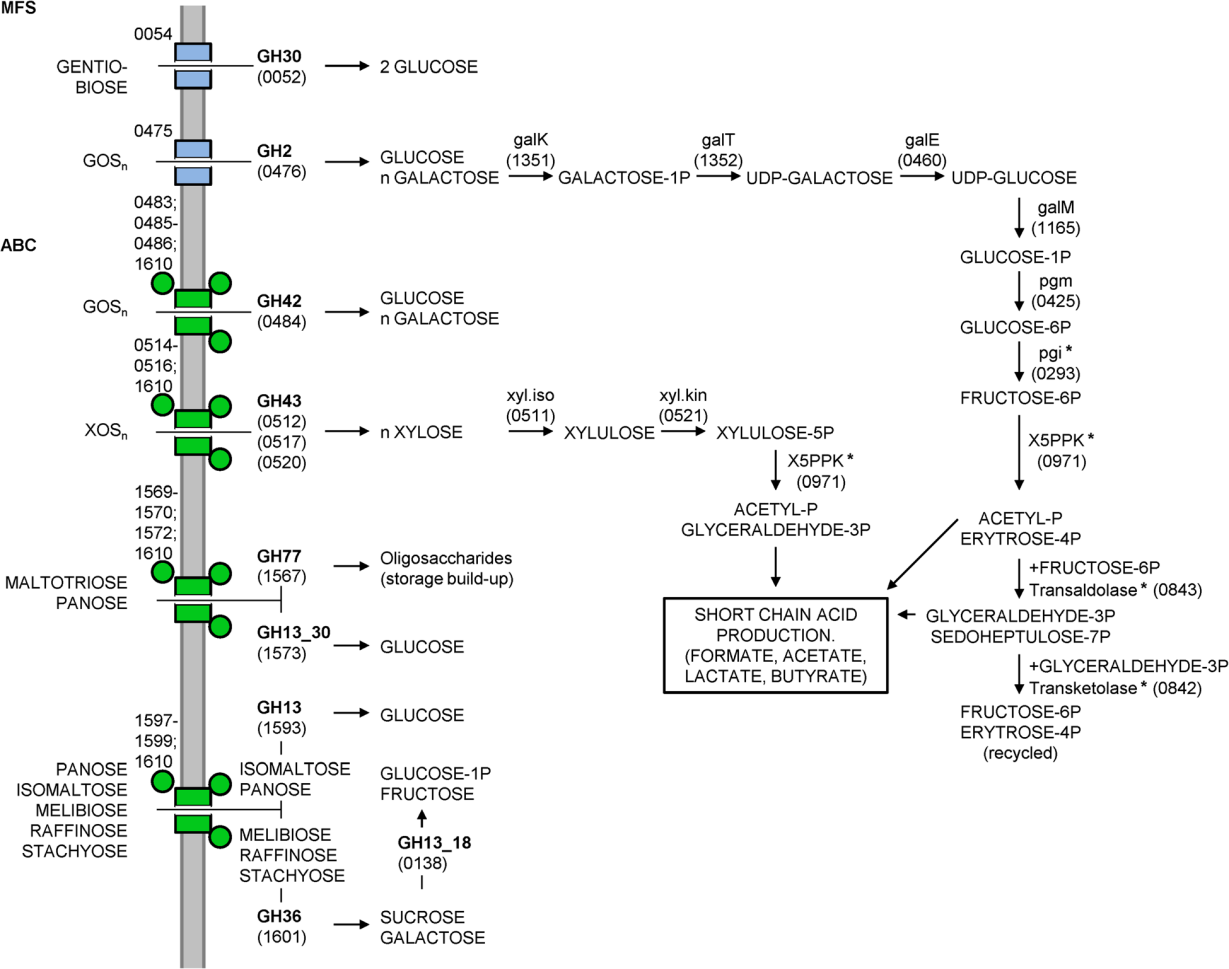
**Proposed pathways for oligosaccharide uptake and catabolism into monosaccharides for entry into the bifid shunt.** Transporters are colored as in Figure 6 and all genes are given by their locus tag. The schematic pathways for glucose (entering as glucose-1P), galactose, fructose (entering as fructose-6P) and xylose are shown with the main steps of the bifid shunt. All constitutive highly expressed genes (Figure 1) are denoted with an asterisk (*)

In summary, all proposed pathways deduced from the identified gene clusters are shown in Figure 7, where potential prebiotic oligosaccharides are internalized and hydrolyzed into products that can be metabolized by the bifid shunt pathway [39]. Consistently, the majority of the bifid shunt genes were found to be highly expressed in all conditions as marked in Figure 7. Notably, a single putative phosphoketolase gene is encoded in *B. lactis* Bl-04, suggesting that this gene product could phosphorolyse both fructose-6P and xylulose-5P as the initial step of the bifid shunt, as previously described within *B. lactis*[46].

### Differentiation of transporter functionalities by transmembrane topology and sequence diversity

To differentiate the functionality of ABC and MFS transporters, the putative α-helical topology of the membrane spanning domains of all predicted oligosaccharide transporters in *B. lactis* Bl-04 was mapped (Table 3). Notably, the gentiobiose-specific MFS transporter (Balac_0054) possesses 11 predicted transmembrane helices, indicating structural-functional divergence from previously identified homologous MFS permeases displaying mainly 12 transmembrane helix topology [47]. Furthermore, one permease protein (Balac_1570) constituting part of the maltotriose upregulated ABC transporter was found to be N-terminally truncated and lacking two helices implicated in heterodimer formation in the permease domain of the maltose ABC transporter from *Escherichia coli*[48]. Comparison to an additional putative *B. lactis* Bl-04 maltose transporter (Balac_1563–1565) and the experimentally verified maltose ABC transporters from *Lactobacillus casei*[42] and *Streptococcus pneumoniae*[49] showed that all harbored the additional two α-helical domain, suggesting the divergence of the maltotriose ABC transporter (Balac_1569, 1570 and 1572) from known maltose ABC transporters.

**Table 3:** **Prediction of α-helical topology within oligosaccharide transporters identified in *B. lactis *Bl-04**

**ORF**	**Predicted substrates**	**Class**	**Predicted TMH**^**1**^	**Sequence length (aa)**
0054	Gentiobiose	MFS	11	384
0139	Sucrose (putative)	MFS	12	537
0475	GOS	GPH homolog of MFS	12	505
1240	FOS (putative)	MFS	12	441
1588	arabinofuranosides (putative)	GPH homolog of MFS	12	481
0485	GOS	ABC	6	326
0486	GOS	ABC	6	322
0515	XOS	ABC	6	352
0516	XOS	ABC	6	289
1563	Maltose (putative)	ABC	6	322
1564	Maltose (putative)	ABC	8	457
1569	Maltotriose	ABC	6	278
1570	Maltotriose	ABC	6	284
1597	RFO + IMO	ABC	6	301
1598	RFO + IMO	ABC	6	330

^1^Transmembrane α-helices (TMH) predicted using the Phobius tool [71].

**Figure 8:**
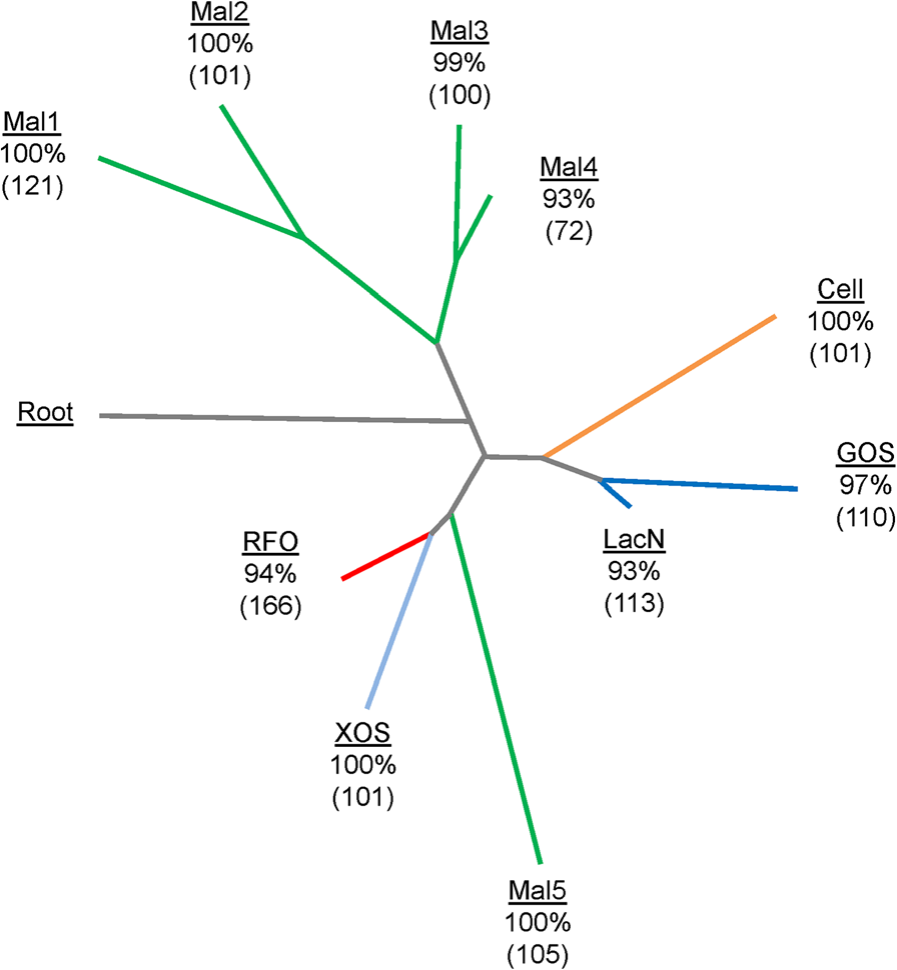
**Functional comparison of the identified oligosaccharide SBPs of ABC transporters in *B. lactis *Bl-04.** The phylogenetic tree was rooted by a characterized fructose-binding protein [69] as a functional and structural outlier group of oligosaccharide-binding proteins [50]. Sub-clusters were defined by bootstrap values in percentages and the characterized SBP(s) identified within each sub-cluster, where the number of sequences are listed in brackets. The tree was colored by substrate specificity (maltose-binding proteins (green), cellodextrins (orange), GOS and lacto-*N*-biose (blue), XOS (light blue) and raffinose family oligosaccharides (red)) and sub-clusters were denoted by numbers as given in Table 4. The raffinose family oligosaccharide cluster was seemingly sub-clustering into two parts but with the current data could not be supported by boot-strap analysis.

Four of the five *in silico* annotated ABC transporters [25] were found to be differentially upregulated, while the remaining putative maltose ABC transporter discussed above (Balac_1563–1565) was found to be constitutively expressed to a comparable level. The transcriptomics data enabled the identification of novel specificities and multiple ligand recognition by the SBPs, recognized as specificity determinants for ABC transporters [50]. This is in agreement with the binding plasticity proposed for ABC-mediated transport [50]. To elaborate on these findings, the phylogenetics of the SBPs were compared to known protein orthologs (Supplemental Table) identified from bifidobacteria and pathogenic GIT-associated bacteria (Figure 8), hence displaying the functional and taxonomical distribution of oligosaccharide SBPs.

## Discussion

Bifidobacteria have been shown to exert a positive influence on the human gut [51] and may selectively utilize oligosaccharides of plant and milk-derived prebiotics [52]. Despite significant advances in bacterial genomics, understanding of carbohydrate uptake and catabolism mechanisms remains elusive, mainly because of poor overall annotation of oligosaccharide transporters where recent advances in uptake of human derived glycans [53] combined with the present study will enable improved functional overview of the *Bifidobacterium* genus with respect to carbohydrate utilization as an important factor for competitive GIT colonization and pathogen inhibition.

### The global transcriptome of *B. lactis* Bl-04

The catabolic adaptation potential of *B. lactis* Bl-04 became apparent from the global comparison of oligosaccharide induced gene expression by principal component analysis (Figure 2). The altered global gene expression by the type of glycoside metabolized (galactoside, glucoside or xyloside) was not influenced by the differentially expressed gene clusters involved in the uptake and catabolism of oligosaccharides. It is likely that global gene expression, induced by carbohydrate source, involves modulation of the metabolic equilibrium within the bacterium. This was observed in *B. longum* for glycoside-induced changes in exopolysaccharide production [54] and the inhibition of enteropathogens by acidification when metabolizing fructose rather than glucose [55]. These findings underscore the effects associated with the catabolism of glycoside type on the overall behavior and potentially probiotic functionality of bifidobacteria in the GIT. Because of the importance for selective utilization of oligosaccharides, we hypothesize a vital role of ABC transporters for prebiotic uptake. Interestingly, a sole oligosaccharide ABC transporter-specific ATP-binding protein was identified in the genome and found to be constitutively highly expressed, consistent with a single ATP-binding protein energizing multiple oligosaccharide ABC transporters as previously described [56]. Likewise, various genes encoding proteins linked to proposed probiotic mechanisms of action, such as adhesion (Balac_1484–1485), phage immunity (Balac_1305) and bile salt hydrolysis (Balac_0863) were found to be highly expressed, supporting the clinically proven probiotic nature of *B. lactis* Bl-04 and reflecting the adaptation to the conditions of the GIT.

Analysis of the differentially expressed genes of *B. lactis* Bl-04 involved in prebiotic utilization revealed upregulation of explicit gene clusters, as was also observed from previous studies of oligosaccharide utilization in probiotic bacteria [35,57]. The uptake of oligosaccharides was facilitated by ABC and MFS types of oligosaccharide transporters, by the lack of phosphoenolpyruvate-dependent phosphotransferase systems in the *B. lactis* Bl-04 genome [25], all associated with glycoside hydrolases.

### Oligosaccharide ABC transporters

The phylogenetic analysis of homologs of identified ABC transporters allowed the assignment of the SBPs identified in the current study into functional clusters harboring experimentally identified counterparts. Within each functional cluster, protein orthologs segregated based on taxonomic distance. Evolutionary adaptation was evident from this analysis as the milk disaccharide lacto-*N*-biose specificity defined by cluster LacN is almost exclusively found within bifidobacteria, while XOS SBP orthologs were dominated by soil bacteria and few GIT associated bacteria with the majority originating from Actinobacteria. This suggests that XOS utilization by bifidobacteria shares a metabolic niche within the GIT with xylan utilizing commensal bacteria [58].

The phylogenetic analysis indicates convergent evolution of a subset of maltose ABC transporter, based on the upregulated maltotriose ABC transporter (Balac_1569, 1570 and 1572). The diversity of canonical maltose SBP orthologs was illustrated by their segregation into four sub-clusters (clusters Mal1–Mal4), where a taxonomical sub-clustering was observed. A distant sub-cluster (Mal5) was defined by the maltotriose upregulated binding protein (Balac_1572). Notably, the corresponding permease domain of this ABC transporter was distinguished from identified maltose specific counterparts by the lack of two N-terminal α-helices (Balac_1570, Table 3), supporting the proposed convergent nature of this type of maltose transporter, which seems to share topological features with the raffinose and XOS type binding proteins (Figure 8).

### Novel specificities of glycoside hydrolases for GIT adaptation

Identification of single gene being differentially upregulated by specific oligosaccharides revealed novel enzyme substrate specificities as compared to the initial *in silico* annotation of the hydrolytic capabilities of *B. lactic* Bl-04 [25]. Interestingly, the observation of a GH42 β-galactosidase being induced by the β-1,6-glucoside gentiobiose was intriguing as only β-galactosidases have been reported in this family. Thus, the GH30_1 putative β-glucosidase is the more likely candidate for gentiobiose hydrolysis. Nonetheless, the transcriptomics data suggests that gentiobiose is specifically transported by the MFS permease, thus defining a novel specificity for this MFS transporter. It remains to be investigated if additional substrates are taken up by the MFS permease including possible substrates for the upregulated GH42, which exhibits modest sequence identity (≈30%) to characterized GH42 enzymes.

No putative glycoside hydrolase was differentially up-regulated on cellobiose. However, transcriptional mining of *B. lactis* Bl-04 identified a constitutively expressed GH1 β-glucosidase (Balac_0151). The β-glucosidase displayed 51% amino acid identity to the GH1 β-glucosidase from *B. brevis* UCC2003 shown to be active on cellobiose and cellodextrin (β-1,4-glucooligosaccharides) initially transported by an ABC transporter [59], supporting the suggested function of Balac_0151. Furthermore, the only transporter differentially up-regulated on cellobiose was the above ABC transporter (Balac_1572) which was also up-regulated by maltotriose (Figure 5). This indicates a potentially dual specificity of the transporter likely to have evolved from multiple sugar metabolism-types of oligosaccharide ABC transporters (Figure 8).

**Figure 9:**
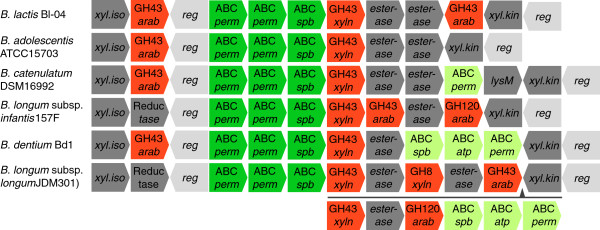
**Genomic content and organization of XOS utilization gene clusters identified within bifidobacteria.** All strains were ordered top down by highest sequence similarity of the XOS-binding proteins to the XOS-binding protein of *B. lactis* Bl-04 (balac_0514). Gene functions are colored as: Glycoside hydrolases (red), XOS ABC transporters (green), xylose ABC transporters (light green), transcriptional regulators (light grey), and putative XOS esterases, xylose isomerases (xyl.iso), xylulose kinases (xyl.kin), alcohol dehydrogenases (Reductase) and a putative secreted amidase (lysM) (all dark gray). All GH43 enzymes were annotated and differentiated by protein similarity to previously characterized xylosidases (xyln) or arabinofuranosidases (arab) together with the GH8 and GH120 enzymes [37,38]. An insertion in *B. longum* JDM301 is shown by below the gene cluster with an arrow indicating the position of the insert.

The uptake and catabolism of XOS within bifidobacteria was recently proposed [37,60]. Comparative genomic of genes involved with XOS utilization within bifidobacteria (Figure 9) reflected a core gene structure of the XOS ABC transporter with a GH43 β-1,4-xylosidase (Balac_0517), while the occurrence of arabino-furanosidases, xylanases of GH8 and GH120 and carbohydrate esterases suggested more species and strain specific adaptation to utilize specific types of XOS *e.g.* arabinosyl decorated fragments. The multiplicity of GH43 arabino-furanosidases reflects the complexity of arabinosyl decorations that occurs naturally in arabinoxylan and its degradation products. Two putative oligosaccharide esterases, distantly related to previously identified xylan acetyl esterases [61] and conserved among bifidobacteria, were upregulated by XOS and xylobiose in *B. lactis* Bl-04. This implicates these putative esterases in de-esterification of xylan fragments transported into bifidobacteria. Taken together, this suggests an exquisite metabolic versatility in the uptake and utilization of xlyan degradation fragments that occur naturally with a diversity of arabinosyl and esterified side chain decorations.

The ABC transporter mediated uptake of GOS coupled with co-induction of a GH42 showed homology to a *B. longum* NCC2705 gene cluster upregulated by lactose [35]. Interestingly this gene cluster diverges from those identified for human milk oligosaccharide uptake [62] both by the similarity of the associated SBP (Figure 8, LacN versus GOS) and the GH encoded in the gene clusters (GH42 versus GH112). Therefore, *B. lactis* Bl-04 has evolved a broad oligosaccharide utilization profile for potential prebiotics and dietary fibers.

## Conclusion

In conclusion, the overall global gene expression of *B. lactis* Bl-04 was dependent of the type of glycoside utilized (galactosides, glucosides or xylosides) potentially linking the prebiotic catabolism of the bacteria to the overall behavior in the GIT. From the transcriptional analyses, we identified the genetic loci encoding MFS and ABC transporters concurrently with glycoside hydrolases for utilization of potential prebiotic oligosaccharides of α- and β-linkages and varying glycoside composition. This highlights the metabolic versatility of *B. lactis* Bl-04 and offers a means of enhancing probiotic effects by dietary supplementation with novel prebiotics. Furthermore, this study provides molecular level support for utilization of potential prebiotics, some of which are already known to be bifidogenic, and paves the way for expanding synbiotic formulations targeting specific groups of probiotic bacteria.

## Methods

### Culture preparation

*B. animalis* subsp. *lactis* Bl-04 (ATCC SD5219) was originally isolated from a human fecal sample [25]. Cultures prepared for transcriptional analysis were propagated in 0.22 μm filtered LABSEM media [63] pretreated by the Hungate method for oxygen removal [64]. The media was supplemented with 1% (w/v) of the 12 tested carbohydrates (Table 1) and each culture was transferred for five passages, under anaerobic conditions, on each carbohydrate before being harvested in the early logarithmic growth phase (*OD*_600_ = 0.3–0.5) by centrifugation at 4°C (3,000 g for 15 min) and flash freezing of the cell pellet for storage.

### RNA isolation and microarray hybridization

Cells were mechanically disrupted by beadbeating and total RNA was isolated using Trizol-chloroform extraction (Invitrogen, Carlsbad, CA). Genomic DNA was removed with Turbo DNAse (Ambion, Austin, TX), followed by RNA purification using a RNeasy Mini Kit (Qiagen Inc., Valencia, CA) [65].

Reverse transcription of total RNA, fragmentation and 3’ biotin labeling of cDNA was done using 10 μg of total RNA in duplicates for each of the 12 conditions and microarray hybridizations were performed using the Affymetrix GeneChip® system (Affymetrix, Santa Clara, CA). Total RNA was reverse transcribed using random primers and SuperScript II Reverse Transcriptase (Invitrogen, Carlsbad, California) and cDNA was purified using MinElute PCR Purification kit (QIAGEN, Inc., Valencia, CA) with a final elution volume of 12 μl. Subsequently, cDNA fragmentation into 50–100 bp was performed using DNase I (GE Healthcare, Waukesha, WI) and cDNA fragments were biotin-labeled using GeneChip DNA labeling reagent (Affymetrix) and terminal deoxynucleotidyl transferase (Promega, Madison, WI).

Labeled cDNA fragments were hybridized at Utah State University using Affymetrix custom-made chips. All extracted data was imported into SAS JMP Genomics (SAS Institute Inc, Cary, NC) before being quantile normalized and modeled using a one-way ANOVA for identification of differentially upregulated genes using a threshold value of α = 0.005 and Bonferroni correction.

The full genome transcriptome (98.1% of the total ORF) for each of the 12 growth conditions was used for the one-way ANOVA analysis. The pairwise analysis was done by comparing each single condition to the other 11 for a total of 66 pairwise conditions. To each of these 66 comparisons, the ANOVA identified a number of genes that were significantly upregulated, for further *in silico* validation by real-time quantative PCR of selected genes. The identified upregulated genes from the ANOVA were then annotated for potential involvement in carbohydrate utilization.

### Real-time quantitative PCR (RT-qPCR) validation of microarray

RT-qPCR was performed on five selected genes (Table 3) found to be differentially upregulated. The DNAse-treated total RNA, identical to the RNA used in microarray sample preparation, was used as template for each of the above 12 growth conditions, measured in triplicates. Experiments were conducted with a QRT-PCR thermal cycler (I-cycler; Bio-Rad, Hercules, CA) in combination with the iScript One-Step RT-PCR Kit with SYBR Green (Biorad).

### Construction of phylogenetic tree of carbohydrate SBPs

The sequence dataset was compiled from oligosaccharide-binding proteins all identified from previous works or from the current study (Table 4). Sequence homologs for each protein entry were identified by BLAST [66] and restricted to either 100 hits or an e-value of 10^-3^ against the non-redundant database. All redundant sequences were removed and the remaining sequences together with a monosaccharide (fructose) binding proteins were aligned with ClustalX [67] using the Blosum series substitution matrix and a gap opening penalty of 2, compared to the standard penalty of 10. The resulting phylogenetic tree file was visualized using Dendroscope [68]. Bootstrap values were calculated by ClustalX using standard conditions (1000 iterations).

**Table 4:** **Identified clusters of oligosaccharide-binding proteins from Figure**8

**Cluster**	**Sub-cluster**	**Substrate specificity**	**Identified Organism**	**Reference**
Malto-oligosaccharides	1	α-(1,4)-gluco-oligosaccharides	*Listeria monocytogenes*	[72]
			*Streptococcus pneumoniae*	[73]
	2	β-Cyclodextrin and maltose	*Bacillus subtilis*	[74]
	3	Maltose	*L. casei* BL23	[42]
	4	Putative maltose	*B. animalis* subsp *lactis* Bl-04	This study
	5	Maltose	*B. longum* NCC2705	[35]
		Maltotriose	*B. lactis* Bl-04	This study
β-glucosides	-	β-(1,4)-gluco-oligosaccharides	*B. breve* UCC2003	[59]
β-galactosides	A	Lactose and	*B. longum* NCC2705	[35] and
		β-galacto-oligosaccharides	*B. lactis* Bl-04	This study
	B	Lacto-*N*-biose	*B. bifidum*	[75]
			*B. longum*	[76]
XOS	-	β-(1,4)-xylo-oligosaccharides	*B. lactis* Bl-04	This study
RFO	A	Raffinose and isomaltose	*Streptococcus mutans*	[43]
	B	Raffinose	*B. longum* NCC2705	[35]
		Raffinose and Isomaltose^1^	*B. lactis* Bl-04	This study
Root	-	Fructose	*B. longum* NCC2705	[69]

Clusters are shown by numbers and if possible sub-clusters are listed with letters. The experimentally identified oligosaccharide-binding proteins used to generate the tree are listed in the corresponding cluster and sub-cluster if possible.

^1^Including melibiose, panose and stachyose.

**Table 5:** Primer pairs used for RT-qPCR

**ORF**	**Primer 5' – 3'**	**Product size (bp)**
0054	CACACTCGCTCGAGATTC	140
	AGGCCAATCATGCATACG	
0475	GCTGACGATGGGAATGAC	160
	GCTCGACGTGTTCTACTC	
0483	CGTCGGAGTTCTTGATGG	142
	CAGGCAGCCTATGACTTC	
0514	GGCTGACCTTGGATTCTT	145
	CTTCTCGCCCATGTAGTTG	
1565	GAACGCCGTAGATCTTGC	148
	ATGTTCGCCAATGACCAG	

### Microarray submission

All raw data have been deposited in the GEO database (Accession number: GSE41906) and complies with the MIAME guidelines.

## Abbreviations

(ANOVA): Analysis of variance; (ABC): ATP-binding cassette; (GOS): β-galacto-oligosaccharides; (COG): Clusters of Orthologous Groups; (GH): Glycoside hydrolase; (MFS): Major Facilitator Superfamily; (RFO): Raffinose family oligosaccharides; (XOS): Xylo-oligosaccharides

## Authors’ contributions

Designed research: RB, MAH, SL, BS, TRKl; Performed research: JMA, YJG; Contributed new reagents (SL) and analytic tools: YJG, TRK; Analyzed data: JMA, RB, YJG, TRK; Wrote the paper: JMA, RB, MAH, SL, YJG, BS, TRK. All authors read and approved the final manuscript.
